# The Effects of School Closures on Influenza Outbreaks and Pandemics: Systematic Review of Simulation Studies

**DOI:** 10.1371/journal.pone.0097297

**Published:** 2014-05-15

**Authors:** Charlotte Jackson, Punam Mangtani, Jeremy Hawker, Babatunde Olowokure, Emilia Vynnycky

**Affiliations:** 1 Department of Infectious Disease Epidemiology, London School of Hygiene and Tropical Medicine, London, United Kingdom; 2 Statistics, Modelling and Economics Department, Public Health England, London, United Kingdom,; 3 Field Epidemiology Services, Public Health England, Birmingham, United Kingdom; 4 West Midlands Public Health England Centre, Public Health England, Birmingham, United Kingdom; Arizona State University, United States of America

## Abstract

**Background:**

School closure is a potential intervention during an influenza pandemic and has been investigated in many modelling studies.

**Objectives:**

To systematically review the effects of school closure on influenza outbreaks as predicted by simulation studies.

**Methods:**

We searched Medline and Embase for relevant modelling studies published by the end of October 2012, and handsearched key journals. We summarised the predicted effects of school closure on the peak and cumulative attack rates and the duration of the epidemic. We investigated how these predictions depended on the basic reproduction number, the timing and duration of closure and the assumed effects of school closures on contact patterns.

**Results:**

School closures were usually predicted to be most effective if they caused large reductions in contact, if transmissibility was low (e.g. a basic reproduction number <2), and if attack rates were higher in children than in adults. The cumulative attack rate was expected to change less than the peak, but quantitative predictions varied (e.g. reductions in the peak were frequently 20–60% but some studies predicted >90% reductions or even increases under certain assumptions). This partly reflected differences in model assumptions, such as those regarding population contact patterns.

**Conclusions:**

Simulation studies suggest that school closure can be a useful control measure during an influenza pandemic, particularly for reducing peak demand on health services. However, it is difficult to accurately quantify the likely benefits. Further studies of the effects of reactive school closures on contact patterns are needed to improve the accuracy of model predictions.

## Introduction

The World Health Organization currently recommends that school closures are considered as part of a mitigation strategy during an influenza pandemic [1]. However, it has been difficult for epidemiologists and public health services to make clear recommendations to policy makers, as the impact of such closures remains unclear [2]–[5]. Recent reviews of the epidemiological evidence have concluded that school closures may have some benefits [3], [5], which should be balanced against the significant social and economic consequences of the intervention [3].

It is difficult to draw conclusions on the effectiveness of school closures from epidemiological data. Observational studies frequently vary in factors studied, such as the timing and duration of closure, case definitions, and population covered [5]. In addition, other interventions have often been used concurrently with school closures. Consequently, mathematical modelling has increasingly been used to predict the effects of school closure on influenza outbreaks. Previous reviews [6], [7] have either summarised the results of a small number of models of school closures during an influenza pandemic [7]–[11] or have examined models of the effects of multiple interventions [12]. Here, we systematically review published work which used simulation modelling to study the effects of school closure to control an influenza pandemic.

## Methods

Medline and Embase were searched in December 2012 (see the supporting information for the full search strategy used in Medline; similar search terms were used in Embase). No date or language limits were applied, although papers in languages other than English were excluded later. To allow for delays in papers being listed in these databases, a broad search of Pubmed (for the words “influenza” and “school”) was also carried out, covering publication dates from 1 August to 31 October 2012.

Relevant papers from the reference lists of the retrieved articles were also identified. Issues of *Eurosurveillance* (23 April 2009 to 25 October 2012), *Morbidity and Mortality Weekly Report* (24 April 2009 to 26 October 2012) and *Emerging Infectious Diseases* (April 2009 to October 2012) were hand searched. Search results were also supplemented with papers from the reviewers' collections.

Studies were included if they modelled school closures during an influenza outbreak and allowed comparison of baseline simulations with no intervention (or a specified intervention) to simulations in which schools were closed. Models of generic “social distancing” were excluded. We excluded epidemiological studies which used modelling techniques to estimate changes in transmission resulting from school closure during particular outbreaks. Such studies (which are included in another review [5]) are useful in informing assumptions made in transmission models, but are beyond the scope of this review of predictive modelling studies. We summarize that work briefly in the discussion.

Abstracts (and full text where necessary) were screened initially by one reviewer; a second reviewer assessed any paper whose usefulness or findings were unclear to the first reviewer. The following information was extracted from the text, tables and/or figures provided in the studies, where available: type of model; population structure and contact rates; infection parameter values (basic reproduction number, infectious and latent periods); threshold for closing schools and duration of closure; assumed effects of school closure on contact patterns; predicted percentage reduction in the peak incidence of infection, defined as 100×((peak in the absence of school closure – peak with school closure)/peak in the absence of school closure); predicted percentage reduction in the cumulative attack rate, defined as 100×((cumulative AR in the absence of school closure – cumulative AR with school closure)/cumulative AR in the absence of school closure); predicted effect on time to the peak of the epidemic; predicted effect on the duration of the epidemic.

We summarised the predicted effects on the peak and cumulative attack rate graphically, for different assumptions about the effects of school closures on contact patterns and the value of the basic reproduction number (R_0_, the average number of secondary infectious individuals generated by a typical infectious individual in a totally susceptible population).

Some of the identified studies presented several estimates of the predicted effects of school closure on measures such as the cumulative attack rate, corresponding to different sets of assumptions (e.g. about the basic reproduction number and the effects of school closures on contact patterns). Where possible in these cases, to illustrate the range of estimates, the most extreme values derived for each value of viral transmissibility were extracted and presented along with the estimate derived from the main analysis.

## Results

1976 papers were identified through Medline and Embase, of which 146 were read in full. 40 of these were eligible for inclusion in the review, as were five from other sources (Figure S1 in File S1). The papers are summarised in Table S1in File S1 and described in detail in Table S2 in File S1.

Most (30/45) of the included studies used individual-based models; a further five used network models and nine compartmental models (Table S2 in File S1; see supporting information in File S1 for definitions of modelling terms). One additional study (referred to as “other” in Table S2) used a household model describing transmission within and between households and in the community and workplaces. All but three of the models were age-structured. The assumed effect of school closures on contact patterns varied between studies and was rarely based on empirical data. Three studies, however, estimated the effects of school closures on contact patterns by fitting the models to incidence data spanning periods during which schools were open and closed [13]–[15], and two further studies used empirical data on contact patterns collected during term time and a school holiday [16], [17]. Most analyses assumed that contact between children (or contact at school) was reduced or eliminated during school closures, whilst contacts made with other age groups or outside school were either unaffected or increased.

### Predicted effects on peak incidence and cumulative attack rates

**Figure 1:**
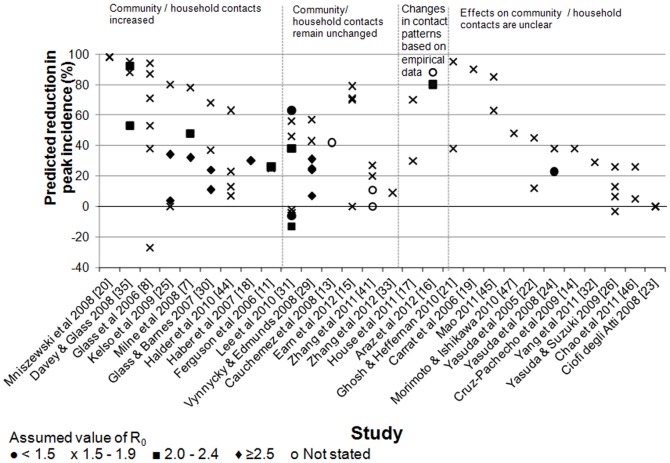
Summary of the estimated effects of school closures on peak incidence of pandemic influenza (all ages) predicted by the modelling studies. Different symbols are used to reflect the assumed value for R_0_. The findings are grouped according to whether they assumed that the community/household contacts increased, remained unchanged, the assumptions about contact were based on empirical data or were unclear. Some studies assumed that workplaces and/or other public places also closed [11], [14], [23]. All studies that stated their assumptions regarding the effects of school closure on contact patterns assumed that contacts between school-aged children were reduced or eliminated.

**Figure 2:**
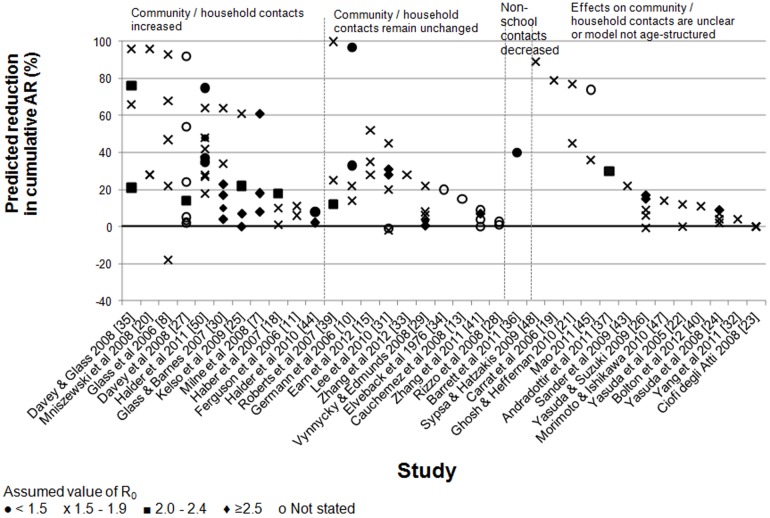
Summary of the estimated effects of school closures on cumulative incidence of pandemic influenza (all ages) predicted by the modelling studies. Different symbols are used to reflect the assumed value for R_0_. The findings are grouped according to whether they assumed that the community/household contacts increased, remained unchanged, the assumptions about contact were based on empirical data or were unclear. Some studies assumed that workplaces and/or other public places also closed [11], [23], [28]. All studies that stated their assumptions regarding the effects of school closure on contact patterns assumed that contacts between school-aged children were reduced or eliminated.

**Figure 3:**
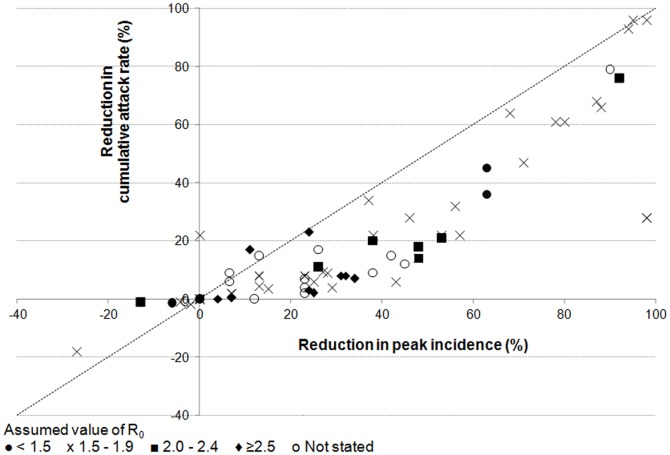
Plot of the predicted reduction in the cumulative attack rate against that in the peak incidence (all ages). Each marker represents the results of one analysis. Different symbols are used to reflect different values for R_0_.

Most modelling analyses indicated that school closures would lead to reductions in the peak incidence and cumulative attack rate (Figures 1 and 2). Predictions of the reduction in the peak incidence were typically 20–60% (e.g. [11], [18]), but some studies predicted much larger reductions of ≥90% [19]–[21]. Reductions in the cumulative AR were usually smaller than those in the peak incidence (Figure 3). Several studies predicted small (<10%) or no reduction in the cumulative AR (e.g.[11], [18], [22]–[33]) whilst a few predicted substantial reductions (e.g. ≥90%) [8], [10], [27], [34], [35]. Only two studies[8], [31] predicted that the peak incidence might increase markedly under some circumstances following school closures, e.g. by 27% if school closures caused a doubling in the number of contacts in the household and community [8], or by 13% if school systems were closed for two weeks at a prevalence of 1% in the general population (and if R_0_ was 2.4) [31]. As the authors discuss, these increases appeared to result from the assumptions that school closure occurred early and briefly [31] or that they resulted in children doubling their numbers of contacts [8]; under other assumptions, both of these studies predicted that school closure would reduce the peak incidence. Of these studies, one predicted that the cumulative AR could increase by 18% [8] whilst the other did not predict substantial increases in the cumulative AR under any of the scenarios reported [31]. One study predicted an overall reduction in the cumulative AR, but an increase of up to 48% in the cumulative AR for adults in some situations [16].

Studies which explored the effects of school closures on age-specific peak incidence or cumulative attack rates typically predicted that the reductions in both were greater for children than for adults [13], [15]–[17], [19], [22], [25], [29], [36]. For example, closing schools at a threshold incidence of 23 cases/100,000/day might reduce peak incidence by 51% in children and 41% in adults, and the cumulative AR by 21% in children and 12% in adults [13]. However, one study (which included a 20% reduction in workplace and community contacts as well as an unspecified reduction in contact between children) predicted the largest reductions in the cumulative AR for middle-aged and older adults (∼40%, compared to a reduction of 22% for schoolchildren) [37]. The reasons why the findings from this paper differ from those of the other papers are unclear, but could have been influenced by the assumed effects of school closures on contact patterns and the baseline transmission probabilities. Another study predicted overall reductions in cumulative ARs if schools were closed, but that the number of infections which occurred in locations other than schools would be higher than during an unmitigated outbreak [32].

The size of the reductions in the peak and cumulative attack rate resulting from school closures depended on four key factors: the basic reproduction number (R­_0_), the effects of school closures on contacts between children, the timing of school closures, and the contact patterns between children before schools were closed. The greatest reductions (especially for the peak attack rate) were usually predicted when R_0_ was relatively low, e.g. <2 [7], [8], [10], [16], [25], [27], [29]–[31], [35], [38], [39], when school closures were assumed to cause large reductions in contact between school-aged children [8], [13], [29], [35], when schools were assumed to close relatively early in the epidemic [13], [18], [25], [27], [30], and when attack rates were higher in children than in adults [7], [8], [30], [34], [40]. For example, one study reported that the peak incidence could be reduced by 78%, 48% and 32% if R_0_ was 1.5, 2.0 or 2.5, respectively [7]. One study was an exception to these generalisations, predicting the greatest reduction in the peak demand for intensive care unit (ICU) beds when R_0_ was high [17]. Also, in several studies, the relationship between the timing of closure and the effects on the cumulative and peak AR was not always simple [16], [33], [41], [42]_ENREF_16 (as discussed below).

A few studies evaluated the potential effects of school closures on hospitalisations and deaths. One study predicted a large reduction in hospitalisations (79%) if schools were closed [19]; another suggested smaller reductions of up to 14% or potentially a slight increase of ∼3%, depending on the threshold for and duration of closure [18]. Another study predicted that peak demand for ICU beds could be reduced by 30–70% by optimally timed school closures [17]. Two studies predicted reductions in deaths of 78% [19] and 23% [43]; another predicted that deaths could decrease by up to ∼17% but could also increase by almost 10%, again depending upon the threshold for and duration of closure [18]. Deaths and hospitalisations were related to the threshold and duration of closure in a less straightforward way than were illness rates in this model, as it assumed that school closure increased transmission in households and the community to individuals outside the school age range, for whom the probabilities of hospitalisation and death given infection were assumed to exceed those among school-aged children [18].

### Predicted effects on the duration of the epidemic

Most models predicted that closing schools would delay the epidemic peak, usually by no more than 1–3 weeks [8], [11], [14], [22], [23], [25], [26], [29], [31]–[33], [44]–[46], but one model suggested that school closure would not affect the timing of the peak [19]. A few studies suggested that school closures could bring the peak forward compared to the unmitigated epidemic [8], [16], [18], [25], [45]. When an earlier peak was predicted, the peak was generally lower and less sharp than in the unmitigated scenario.

Increases in the duration of the epidemic of 1–3 weeks were commonly predicted [14], [18], [22], [25], [30]–[32], with some models predicting increases of about a month [15], [19], [24], [45], [47] or more [8], [29]. Four studies suggested that school closures could shorten the epidemic (by 11 days [48], 2–3 weeks [8], [36], ∼1–3 months [16]), whilst another found little effect on the duration [26].

Again, these predictions depended on assumptions about R_0_, the reduction in contact resulting from school closures, the threshold incidence for school closure, and the extent to which attack rates were age-dependent. For example, high values of R_0_ were commonly associated with the smallest effects on the timing of the peak [11], [29]–[31] and the duration of the epidemic [25], [29], [30].

### Predicted effects of the duration of and threshold for school closure

**Figure 4:**
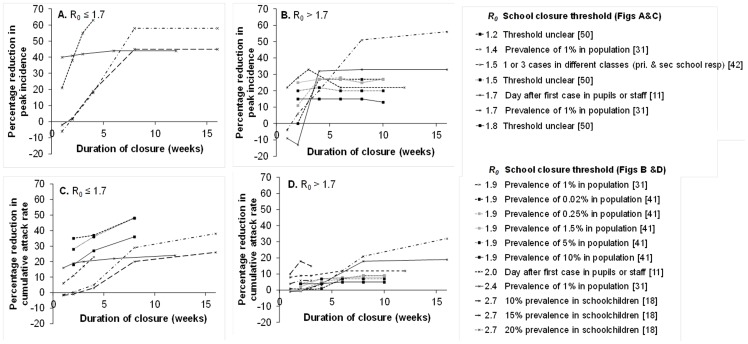
Influence of the duration of school closure on the predicted effects on pandemic influenza. Reductions in peak incidence (A and B) and cumulative attack rates (C and D) for different values of R_0_ and assumed thresholds for school closure. Lines join predictions from the same model using the same sets of assumptions.

Several studies explored the effect of the duration of school closure on the peak and/or cumulative incidence [11], [14], [16], [18], [22], [26], [31], [33], [34], [41], [42], [44], [49], [50]. Of these, eight modelled different durations of closure measured in weeks [11], [16], [18], [31], [33], [41], [44], [50] (Figure 4); one modelled durations of closure ranging from 4–7 days [26] and five compared temporary closures (of 7–60 days) with permanent closures [14], [22], [34], [49], [50] (i.e. once closed, schools did not reopen during the time period modelled).

Several studies reported that the impact of school closures increased with the duration of closure (Figure 4), although increasing the duration above 8 weeks generally had little extra benefit. One study suggested that peak and cumulative attack rates could increase slightly if schools were closed for two weeks or less [31], but the other studies shown in Figure 4 did not predict such increases [11], [18], [44], [50]. Early closures were also often associated with the greatest reductions in peak and cumulative ARs. For example, if schools were closed when incidence exceeded 100 cases/100,000/day, the peak incidence might be reduced by 42%, but the reduction would be only 21% if the threshold was 1000 cases/100,000/day [13].

However, several analyses suggested that the effect of school closures depended on both the duration of closure and the time (or incidence) at which schools were closed [16], [33], [41], [42]. These studies often reported that closing schools at an intermediate threshold was more effective than closing either very early or very late. For example, one study found that the effects on the peak AR were insensitive to the threshold prevalence for closure as long as it was ≤1.5% and the duration of closure was ≥4 weeks; above this closure threshold, the benefit of school closure decreased as the threshold increased [41]. In another study, intermediately timed closures were again more beneficial than very early or very late closures; the effect was most marked for long closures and low R_0_ [42]. A third study found that the optimum threshold for closure depended on the duration, e.g. if schools were closed for <8 weeks then the higher the threshold, the lower the cumulative AR (incidence thresholds up to 5% were investigated), whereas if closure lasted longer, a lower threshold (e.g. 1.5%) was optimum [33].

One study investigated the age-specific effects of varying the threshold and duration of closure (Figure S2 in File S1) [16]. In this study, assumptions about contact patterns and how these were affected by school closure were derived from empirical contact data [51]. For R_0_ between 1.1 and 1.5, closures lasting ≤4 weeks led to increases in adult ARs but decreases amongst children; the benefit to children increased, and the harm to adults decreased, as the duration of closure increased. For closures lasting 4–12 weeks, the benefits of school closure increased with duration for both children and adults, but increasing the duration of closure above 12 weeks had little extra benefit. For closures lasting 4 weeks or less, the threshold made little difference but for longer closures, closing schools when prevalence in school-aged children was low was more effective than waiting until prevalence was higher. The benefits of school closure were greater if R_0_ was assumed to be 1.1–1.5 compared to 1.5–2.1, particularly for adults. In the higher transmissibility scenario, the cumulative AR in adults increased for all closure durations ≤12 weeks unless the threshold prevalence was ≥2%.

The maximum threshold at which school closure can occur and still be beneficial is unclear. One study estimated that, in the scenario where school closures were most effective (low R_0_ and attack rates higher in children than adults), the attack rate would be similar to that in the unmitigated scenario if closure was delayed until the prevalence of infection in children was 20% [30].

All five studies which compared temporary and permanent closures predicted the greatest reductions in peak and/or cumulative attack rates with permanent closure [14], [22], [34], [49], [50]. One study argued that the duration of closure was more important than the closure threshold in determining the effect on the epidemic, and that schools should close for at least eight weeks [31]. Some studies predicted reasonably large effects with shorter closures than this, e.g. reductions of 38% [44] or 41% [11] in the peak incidence if closure lasted for two weeks. One further study estimated the effects of closing schools for 4–7 days; in this model, the benefit increased with duration of closure even over this limited range (e.g. the cumulative attack rate was almost unaffected by a four-day closure but was reduced by 15% if schools were closed for 7 days) [26].

The question of when schools should reopen has been addressed in detail in one modelling analysis [35]. This suggested that the threshold for reopening schools determined whether the epidemic recurred: the higher the threshold incidence for reopening, the higher the probability of recurrence, potentially resulting in multiple epidemic peaks. Another modelling analysis suggested that the benefit of closing schools was not reduced substantially as long as the prevalence of infection in children was <1% when schools reopened [30].

### Predicted effects of different school closure strategies

It is unclear from the modelling studies whether there is any difference in effectiveness between closure of individual schools, multiple schools in a local area, or all schools nationally. One study suggested that a policy of “area closure,” in which all schools within 10km of a case closed for a fixed period, produced similar results to a policy in which each school closed following a case in that school [11]. Similarly, another study found no consistent differences between the effects of closing individual schools and closing an entire school system [31], although two others suggested that closing individual schools would be more effective than closing all schools simultaneously [44], [46]. A slightly different situation, in which some communities closed schools while neighbouring communities did not, and mixing between these communities occurred, reduced the effectiveness of school closure [35].

Overall results are summarised in Table 1.

**Table 1:** Summary of the key findings of factors influencing the impact of school closures, as reflected by the predicted reduction in the peak incidence and the cumulative attack rate.

Parameter/scenario	Predicted influence on impact of school closures (assuming that factors other than those specified remain unchanged)
R_0_	Over the range of values of R_0_ investigated in the studies (up to approximately R_0_ = 3.5), the higher the value of R_0_, the smaller the effect of school closure
Age-specific attack rates	School closure is more effective if baseline attack rates are higher amongst children than amongst adults, than if baseline attack rates among children equal or are smaller than those among adults
Effect of school closures on contact patterns	The greater the reduction in contact resulting from school closure, the greater the effect of the intervention *
Timing and duration of closure	
Individual versus area school closures	Results differed between models
Age-specific effects	The effect of school closures is greater on incidence amongst children than that amongst adults
Effect on peak compared to cumulative attack rate	School closures have a greater effect on the peak attack rate than on the cumulative attack rate

* Some ineligible studies suggest that very large reductions in contact may be less beneficial than smaller reductions [60], [61].

## Discussion

Published mathematical models have reached a variety of conclusions about the effects of school closures on the course of influenza outbreaks. Although the predicted reduction in the peak incidence was typically 20–60%, reductions of >90% and an increase of 27% were also predicted, depending on the model assumptions including those relating to contact patterns. Predicted effects on the cumulative attack rate were consistently smaller than those on the peak incidence (e.g. 0–40%) but were also variable: the predicted effects on the peak incidence ranged from reductions of >90% to an increase of 18%, although most studies predicted reductions in both the cumulative and peak attack rates.

Epidemiological studies have estimated that school closures have reduced the total number of cases of pandemic influenza by 28%, 35% and 52% in Calgary, Edmonton, and the province of Alberta, Canada [15]. Routine school holidays in France have been estimated to prevent 16–18% of seasonal influenza cases, with a larger effect on children (18–21% of cases prevented) than adults (14–17%) [13]. These results are towards the lower end of those predicted by the simulation studies, and the relative effects on adults and children are consistent with the model predictions. The experience of the 2009 pandemic in the UK illustrated that school closures (in this case, school holidays) may lead to a reduction in incidence which rebounds when schools reopen [52]. Some of the reviewed modelling papers predicted that school closures would result in such bimodal epidemics, although in others the simulations ended before schools reopened.

Despite the marked quantitative differences in the model estimates, some qualitative results were consistent across many studies. For example, the reduction in peak incidence was consistently predicted to be larger than that in the cumulative attack rate, since the reduction in contact resulting from school closure slows, rather than eliminates, transmission. Even if the effect of school closures on the final size of the epidemic were small, a reduction and delay in the peak incidence may still be achieved. Such a reduction in the peak burden on health services could be highly beneficial if demand for intensive care and other services is high, as seen during the 2009 pandemic [53]–[55]. The slowing of the epidemic, and the delay in the peak, which may result from school closure mean that the intervention may be a useful short term measure to limit transmission whilst a specific vaccine is developed. This may be a more attainable goal of school closure than a reduction in the cumulative attack rate.

School closures are usually expected to be more effective at reducing transmission if R_0_ is relatively low, since the reductions in contact resulting from school closures may then be sufficient to reduce the effective reproduction number to below one. If R_0_ is high, the same reductions in contact may not be sufficient for this to occur. R_0_ in previous pandemics has typically been estimated as approximately 1·5–3·0, but usually less than 2·0 [29], [56]–[58].The predicted effects of school closure are also expected to be greatest if age-specific attack rates are higher in children than in adults (or if contact intensity in schools is high). This is consistent with conditions obtained using an SIR model for identifying priority target groups for interventions, which found that social distancing measures are most effective when targeted at the age group with the highest incidence of infection (which is likely to change over time) [59]. The benefits of school closure are predicted to increase with the assumed reduction in contact. In general, long closures are predicted to lead to the greatest reductions in the peak and cumulative attack rates, although increasing the duration of closure above 8 weeks had little extra benefit.

The optimum timing of school closure was predicted to depend on its duration, although very late closures were consistently found to be relatively ineffective. For example, for short closures, closing schools very early may have less effect than closing them later [16], [33], [41]. This has been attributed to resumption of mixing between susceptible children when schools reopen while influenza is still circulating, allowing them to acquire and transmit infection [31]. For a similar reason, whilst the included studies typically found that the benefits of school closure were greatest when the intervention was assumed to reduce contacts by a large amount, this may not always be the case: studies which used SIR models to investigate the effects of reductions in transmission arising from interventions such as social distancing found that a temporary intervention which caused a small reduction in transmission could reduce the peak and cumulative attack rates by a greater amount than one in which the reduction in transmission was large [60], [61].

Many of the parameters in the identified models were consistent with those estimated for previous pandemics or seasonal influenza. For example, age-specific attack rates and R_0_ were often similar to those from previous pandemics (with sensitivity analyses to reflect the fact that a future pandemic may differ from previous outbreaks). The pre-infectious and infectious periods, the degree of infectiousness over time, and the serial interval, were often based on (or consistent with) data from household studies [9], [11], [19], [29], other transmission studies [9], [11], [30], [46] or data on virus shedding [8], [29], [49]. The individual-based models often utilised detailed data on population characteristics, such as household sizes and age structure, commuting distances and frequency of airline travel, to allow detailed prediction of the spatiotemporal spread of infection [7], [9]–[11], [23], [25], [37], [42], [44], [47], [50]. Differences between models in any of these parameters could contribute to the differences in their predictions. Development of a consensus “baseline” scenario, in which the natural history and behavioural parameters were set at agreed values and which models could use in simulating outbreaks in the absence of interventions, could help to facilitate comparison of results from different models (for example, to assess the roles of differences in household size or travel patterns).

Although many of the models' assumptions relating to the natural history of influenza and human population structure were based on empirical data, a range of assumptions have been made regarding population contact patterns. This is an important limitation of much of the published literature, as predictions of the effects of school closure depend upon the amount of contact (and therefore transmission) between individuals whilst schools are closed and while they are open. For example, it has been estimated that if such contacts increased 1·5 times more during a pandemic closure than during school holidays, then the benefits of school closure would be minimal [13]. This limitation arises partly because there are relatively few data regarding the effects of school closures, particularly reactive closures, on contact patterns [51], [52], [62]–[65] (although several studies estimated the effects of school closures on contact patterns using either empirical data or modelling techniques [13]–[17]). Existing data typically refer to face-to-face conversational contacts, which appear to be a good proxy for transmission of respiratory infections including influenza [66], although further studies are needed of the precise nature of contacts which are sufficient to allow transmission [67]. These contact studies have shown that routine school closures can reduce contact rates substantially, with corresponding reductions in R_0_ if a pathogen emerges while contact patterns resemble those observed during school holidays as compared to term time [63], [68].

Few studies of contact patterns during reactive school closures have been published [64], [69] and the differences between contact patterns during routine and reactive closures remain unclear. Changes in contact patterns during reactive closures may depend on various factors, including the perceived severity of the infection and messages from public health authorities. Despite this uncertainty, relatively few models have explored the effects of different assumptions regarding the effects of school closures on contact patterns.

Most of the modelling studies assumed that school closure would reduce contacts between children, with or without affecting other contacts. Contact studies have found that school closures reduce contact between children substantially but have much less effect on adults' contact behaviour [51], [63], [64]. In a UK study, children's contacts were reduced overall during school holidays, but the number of contacts they made with adults increased [62]. The assumptions made in the modelling studies were therefore generally consistent with empirical data, but incorporating additional contact data into transmission models, as they become available, may increase the reliability of model predictions. Incorporating contact data into transmission models, including models of school closure, has become increasingly common [17], [52], [63], [70]. In the future, it will be important to collect contact data from a variety of settings [71], [72] and to base models on these data, to assess the consistency of contact patterns (and the effects of school closures) and predictions across locations. It will also be useful to collect contact data alongside data on the epidemic curve in the same setting. Notably, the only study which predicted substantial increases in both the peak and cumulative attack rates did so only if school closure was assumed to result in increased contact between schoolchildren [8], which is inconsistent with findings to date from published contact studies; it therefore appears unlikely that school closures would dramatically increase attack rates.

Contact studies also highlight the fact that the number of contacts made and the effects of school closure on these contacts vary substantially with age [51], [63], [64]. Three of the models [14], [39], [40] were not age-structured so were not able to capture this age dependence.

This systematic review included papers published before the end of October 2012. Several simulation studies published since then meet the inclusion criteria [73]–[75] but do not affect the conclusions of the review; the reductions in the cumulative attack rate were variable (ranging from minimal effect [75] to a 50% reduction [74], although timing and duration of closure also varied) and the assumptions about the effects of school closures on contact patterns were not based on empirical data. A further paper assessed the impact of the timing and duration of school closure on its effectiveness as a mitigation strategy, basing its assumptions about contact patterns (and the impact of school holidays on contact patterns) on data collected from an internet-based cohort study conducted in the UK [61]. This study found that the optimum timing for minimising the peak incidence is earlier than that for minimising the final outbreak size.

Several studies have estimated the extent to which school closures during specific pandemic or seasonal outbreaks may have affected contact patterns [13], [15], [76], [77]. These studies were not eligible for this review of predictive modelling of the effects of school closures under different epidemiological conditions, but are included in a separate review of epidemiological studies of school closures [5]. These studies report on events during particular outbreaks without making assumptions about individuals' behaviour or the properties of the causative virus. They therefore provide valuable insights into the effects of school closures on contact patterns and transmission. However, predictive modelling studies such as those summarised in this review are able to investigate which factors (e.g. R_0_, individuals' compliance with social distancing advice) influence the effectiveness of a school closure policy. The two sources of evidence – epidemiological studies and simulation studies – are therefore complementary.

Although papers in languages other than English were excluded from this review, the titles and abstracts (where available in English) were screened and found not to be relevant in all but one case [78].

Overall, modelling work suggests that school closures may be beneficial in reducing peak and cumulative attack rates during an influenza pandemic. Results from models which have used a variety of different assumptions and approaches suggest that this intervention can lead to reductions of 20–60% in the peak incidence of an epidemic and smaller (0–40%) reductions in the size of the epidemic. The size of the reductions are expected to be greater if the transmissibility of the virus is relatively low (e.g. R_0_<2) and if attack rates are higher in children than in adults. These factors should ideally be considered when deciding whether schools should be closed during a pandemic. Further empirical studies of the effect of school closures on contact patterns from different settings are needed for an improved understanding of the potential impact of school closures on the size of a pandemic, as well as modelling studies to assess the sensitivity of predictions to these assumptions. Additionally, other issues not reviewed here should be considered in deciding whether or not to close schools, such as the severity of infection and medical services' ability to cope with excess demand.

## Supporting Information

Information S1 Contains the following Figures and Tables: Figure S1: Identification of mathematical modelling studies of the effects of school closure on influenza outbreak. Figure S2: Further estimates of the influence of the duration of school closure on the predicted effects of pandemic influenza. Table S1: Features of modelling studies identified. Table S2: Mathematical modelling studies of the effects of school closure on pandemic influenza.
(DOCX)

Checklist S1 **PRISMA Checklist.**
(DOC)
