# Supplementary material for: The Effects of School Closures on Influenza Outbreaks and Pandemics: Systematic Review of Simulation Studies
Source: PLoS One. 2014 May 15;9(5):e97297. doi: 10.1371/journal.pone.0097297 (PMC4022492; doi:10.1371/journal.pone.0097297)
Supplement: Information S1 — Contains the following Figures and Tables: Figure S1: Identification of mathematical modelling studies of the effects of school closure on influenza outbreak. Figure S2: Further estimates of the influence of the duration of school closure on the predicted effects of pandemic influenza. Table S1: Features of modelling studies identified. Table S2: Mathematical modelling studies of the effects of school closure on pandemic influenza. (DOCX) [file pone.0097297.s001.docx]

**The effects of school closures on influenza outbreaks and pandemics: systematic review of mathematical modelling studies**

Supporting Information

Search strategy used in Medline

1. ((school adj5 clos*) or (nurser* adj5 clos*) or (kindergarten adj5 clos*) or (daycare adj5 clos*) or (day adj care adj5 clos*) or (preschool* adj5 clos*) or (pre-school* adj5 clos*) or (child* adj5 home) or (schoolchild* adj5 home) or (teenage* adj5 home) or (preschool* adj5 home) or (pre-school* adj5 home) or (adolescent adj5 home) or (social adj5 distanc*)).mp. [mp=title, original title, abstract, name of substance word, subject heading word, unique identifier]

2. exp Schools/

3. 1 or 2

4. (infect* or communicable or contagio*).mp. [mp=title, original title, abstract, name of substance word, subject heading word, unique identifier]

5. (mortality or (attack adj5 rate) or morbidity or incidence).mp. [mp=title, original title, abstract, name of substance word, subject heading word, unique identifier]

6. exp Incidence/

7. exp Infection/

8. exp Communicable Diseases/ or exp Communicable Disease Control/ or exp Communicable Diseases, Emerging/

9. exp Disease outbreaks/

10. 4 or 5 or 6 or 7 or 8 or 9

11. (model* or (mathematical adj5 model*) or (transmission adj5 model*) or (simulation adj5 model*) or (statistical adj5 model*) or (epidemi* adj5 model*) or (dynamic* adj5 model*) or (computer adj5 model*) or simulation).mp. [mp=title, original title, abstract, name of substance word, subject heading word, unique identifier]

12. exp Models, Theoretical/

13. exp Models, Statistical/

14. exp Computer simulation/

15. 11 or 12 or 13 or 14

16. 3 and 10 and 15

**Definition of modelling terms**

*Compartmental models* stratify individuals into different categories (“compartments”) and are usually described using the compartments included. For example, SEIR models include compartments for those who are susceptible to infection (S), infected but not yet infectious (E), infectious (I), and recovered (R). Such models may be deterministic, in which case they describe what happens on average in the population, or stochastic, i.e. allowing for random events to influence the course of the epidemic. In compartmental models, individual members of the population are not followed, but the number of individuals in each compartment is tracked over time.

*Individual-based models* explicitly follow each individual in the modelled population; each person can be assigned characteristics such as age and employment status which determine (amongst other things) their interactions with other people and therefore their probability of becoming infected.

*Network models* are individual-based in that each person is explicitly tracked, but transmission of infection can occur only along links in a pre-specified network of contacts.

*Matrices of “who acquires infection from whom” (WAIFW matrices)* describe the rate at which individuals in different groups (e.g. age groups) come into effective contact in a modelled population. An *effective contact* is defined as one that is sufficient to lead to transmission if it occurs between a susceptible and an infectious individual.

*Assortativity* refers to the extent to which different groups (e.g. age groups) in a modelled population contact each other. If mixing is highly assortative, individuals are more likely to contact others within their own group than they are to contact individuals in other groups. If mixing is disassortative, individuals are more likely to contact individuals in different groups than they are to contact individuals in their own group.

**Figure S1: Identification of mathematical modelling studies of the effects of school closure on influenza outbreak.**

Unique search results

**1976**

*Exclusions (****1830****)*

**1718** based on title and / or abstract

**112** in languages other than English

*Exclusions (****106****)*

**14** studies which modelled generic “social distancing”

**6** reviews / summaries

**53** studies with no analysis of effects of closure on incidence / transmission

**5** studies which did not (specifically) model influenza

**3** studies with no straightforward comparison scenario

**25** epidemiological or contact studies / studies without predictive modelling

*Additions (****5****)*

**1** from reference lists

**1** from handsearching

**2** from supplementary search of

Pubmed

**1** from collections

**Included in review**

**45**

Full text reviewed

**146**

**Figure S2: Further estimates of the influence of the duration of school closure on the predicted effects of pandemic influenza.** Predicted reductions in age-specific cumulative attack rates under A) low transmission (R_0_ = 1.1 to 1.5) and B) high transmission (R_0_ = 1.5 to 2.1) scenarios. Source: Araz et al [^1^](#_ENREF_1).

A

B

**Table S1:** Features of modelling studies identified.

|  | **Number of papers** |
| --- | --- |
| ***Total papers*** | **46** |
| **Type of model** | |
| Individual-based | 30 |
| Network | 5 |
| Compartmental | 9 |
| Other | 1 |
| **Baseline age-specific ARs** | |
| Higher in children than in adults | 27 |
| Relatively uniform with age (e.g. based on 1968 pandemic) | 3 |
| Not age-structured | 3 |
| Age-specific ARs not given or basis not stated | 15 |
| **Threshold for closing schools*** | |
| Pre-emptively or immediately at start of pandemic | 6 |
| Based on case numbers or incidence of infection (e.g. 50 cases per 100,000 population) | 16 |
| Based on cumulative incidence of infection | 6 |
| Based on prevalence of infection | 8 |
| Based on time since beginning of pandemic or local epidemic (e.g. 2-8 weeks) | 12 |
| Unclear / not stated / based on observed timing | 4 |
| **Assumed effects of school closures on contact rates*** | |
| Child-to-child or school-related contact reduced or eliminated, no effects on other contacts | 15 |
| Child-to-child or school-related contact reduced or eliminated, other contacts increased | 11 |
| Child-to-child or school-related contact reduced, other contacts increased or decreased depending on location | 2 |
| Complex changes based on empirical contact data | 2 |
| Uniform reduction in contact rates (model not age-structured) | 3 |
| Not stated / unclear | 12 |
| **Basis of assumptions regarding effects of school closures on contact rates** | |
| Empirically measured contact rates | 2 |
| Fitting of model to incidence data | 3 |
| Other quantitative data | 1 |
| No empirical basis stated | 40 |
| **Information provided on effects of school closure on:** | |
| Cumulative AR | 40 |
| Peak incidence of infection (or peak prevalence) | 33 |
| Time course of epidemic (duration and / or time to peak) | 32 |
| **Sensitivity analysis / exploration of different values of:** | |
| R_0_ | 24 |
| Baseline contact rates | 6 |
| Changes in contact rates associated with school closure | 6 |
| Patterns of age-specific ARs | 6 |
| Threshold for closing schools (measured as incidence or time since start of epidemic) | 19 |
| Duration of closure | 16 |
| Threshold for re-opening schools | 3 |

*Each paper may explore more than one assumption

**Table S2:** Mathematical modelling studies of the effects of school closure on pandemic influenza

| Population structure and contact rates | Infection parameter values | Threshold for closing schools and duration of closure | Assumed effects of school closure on contact patterns | Predicted effect on: | | | |
| --- | --- | --- | --- | --- | --- | --- | --- |
|  |  |  |  | Peak incidence of infection | Cumulative AR | Time to peak | Duration of epidemic |
| **Individual based models** | | | | | | | |
| Elveback et al (1976) [^2^](#_ENREF_2) | | | | | | | |
| Hypothetical population structured to resemble a suburban US community. Age / location-specific contact rates chosen to produce age-specific ARs similar to those for the 1957 and 1968 pandemics. Contact rate greatest in playgroups, then family, then schools (1957) or neighbourhood clusters (1968), then neighbourhood clusters (1957) or schools (1968), then community. | Average latent period = 1.9 days  Average infectious period = 4.1 days  Baseline clinical ARs:  1957 – 35.4% (preschool) 61.8% (school), 23.4% (young adult), 13.1% (older adult), 35.1% (overall)  1968 – 35.2% (preschool) 35.7% (school), 32.1% (young adult), 30.4% (older adult), 33.3% (overall) | Schools either never opened following routine closure, or closed for the second week of the outbreak | Elimination of transmission in schools; no effect on other contact rates.  No empirical basis stated for these assumptions. | NA | Reduced by 90% (using contact rates based on Asian influenza) or 27% (using contact rates based on Hong Kong influenza) if schools never opened, or by 20% with one week closure (Asian influenza) | NA | Not quantified, but stated that the effect of several days’ closure during an outbreak of Asian influenza is greater than that on the cumulative AR. |
|  | | | | | | | |
|  | | | | | | | |
|  | | | | | | | |
| Ferguson et al (2005) [^3^](#_ENREF_3) | | | | | | | |
| Population based on Thai census (2000) and related data. Probability of contact sufficient for transmission maximum in schools, half this value in households and workplaces, and ~1/6 of this value in community.  Schools and workplaces closed at the same time as antiviral prophylaxis is provided. Focuses on eliminating a pandemic at source. | R_0_=1.1 – 2.0  Serial interval = 2.6 days, based on household study of seasonal influenza in 2000.  Baseline clinical AR 17% if R_0_ = 1.5, 25% if R_0_ = 1.8.. | Closure of 90% of schools and 50% of workplaces within 5km of a detected case , for 3 weeks. | Complete elimination of mixing in schools and workplaces. 100% increase in contact in households, and 50% increase in random contacts for individuals withdrawn from school / work.  No empirical basis stated for these assumptions, but chosen to be conservative. | NA | >90% chance of eliminating epidemic (i.e. “preventing a large outbreak (which would eventually lead to a global pandemic)”) if R_0_≤1.7 | NA | NA |
| Yasuda et al (2005) [^4^](#_ENREF_4) | | | | | | | |
| Basis of population structure unclear. Contact rates based on time use data from 30000 individuals in Japan, collected at an unspecified time. | Latent period = 3 days  “Period of infection” = 7 days  Baseline AR not provided | Four days after start of outbreak; closure either maintained for duration of outbreak or reopened after 13 days. | Unclear; presumably complete elimination of contacts at school with no effect on other contacts.  No empirical basis stated for these assumptions. | Reduced by ~45% (permanent closure) or ~12% (13 day closure) | Reduced by 12% (10% in adults, 17% in children, permanent closure) or essentially unchanged (13 day closure) | Increased by ~25% from 20 to 25 days (permanent closure) or ~35% from 20 to 27 days (13 day closure) | Increased by ~40% from 50 to 70 days (permanent closure) or ~20% from 50 to 60 days (13 day closure) |
|  | | | | | | | |
| Ferguson et al (2006) [^5^](#_ENREF_5) | | | | | | | |
| Model as in [^3^](#_ENREF_3) applied to population based on Great Britain / United States census data. Model incorporates simultaneous closure of schools and workplaces. | R_0_ = 1.7 or 2.0  Serial interval = 2.6 days, based on same data as [^3^](#_ENREF_3).  Sensitivity analysis used latent period = 1.2 days and infectious period = 4.1 days, and found that this reduced the impact of interventions.  Baseline clinical ARs 28% (R_0_ = 1.7) or 34% (R_0_ = 2.0) | Each school and 10% (or 50%) of workplaces close from the day after detection of the first case in pupils or staff until up to 3 weeks after the last case in that school. Schools / workplaces can close repeatedly during the pandemic.  Sensitivity analyses explored varying the threshold and duration of closure, and an alternative strategy of “area closure” in which all schools within 10km of a case close. | Elimination of transmission in schools and workplaces. 50% increase in contact rates in affected households; 25% increase in community contacts of affected individuals.  No empirical basis stated for these assumptions.  Sensitivity analysis increased household contacts by 70% but the predicted effects of school closures were “relatively insensitive” to this change. | Decreased by 25-33%, depending on R_0_ and, much less importantly, on the proportion of workplaces closing. Duration of closure has little effect. Number of cases which triggers closure of each school has relatively little effect as long as it is <5.  Similar results were obtained when area closure was introduced. | If R_0_=2.0, decreased by 6-9% (from 34% to 32% or 31%, depending on proportion of workplaces closing).  If R_0_=1.7, decreased by 11-15% (from 27% to 23-24%, depending on proportion of workplaces closing).  Longer closures were associated with slightly increased reductions.  Number of cases in each school which triggers closure of that school has relatively little effect as long as it is <5.  Similar results with area closure. | Delayed by 9-16 days, depending on R_0_ and the proportion of workplaces closing. | NA |
|  | | | | | | | |
|  | | | | | | | |
| Germann et al (2006) [^6^](#_ENREF_6) | | | | | | | |
| Population based on US census data (2000). Contact probability in household > preschool > schools and household clusters > neighbourhood and community.  Model incorporates simultaneous closure of schools, preschools and playgroups. | R_0_= 1.6 – 2.4  Mean latent period = 1.2 days  Mean incubation period = 1.9 days  Mean infectious period = 4.1 days  Serial interval = 3.5 days  Baseline AR 33-54% depending on R_0._ Age-specific attack rate pattern chosen to be in between those of 1957 and 1968. For R_0_ = 1.6, clinical AR 35% (0-4 years) 50% (5-18 years), 27% (19-29 years), 28% (30-64 years), 24% (>64 years), 33% (overall). | All schools in the country closed seven days after pandemic alert, which occurs when 10,000 symptomatic cases have occurred nationwide (corresponding to a cumulative incidence of 3.6 / 100,000). | Elimination of mixing in school-related groups. No effect on other contact rates. Schools remain closed for the duration of the pandemic.  No empirical basis stated for these assumptions. | NA | Predicted reduction ranged from 14% (if R_0_ = 2.4) to 97% (if R_0_ = 1.6) | Not quantified, but stated that social distancing policies slow pandemic spread | NA |
|  | | | | | | | |
|  | | | | | | | |
|  | | | | | | | |
|  | | | | | | | |
|  | | | | | | | |
|  | | | | | | | |
|  | | | | | | | |
|  | | | | | | | |
|  | | | | | | | |
|  | | | | | | | |
|  | | | | | | | |
| Haber et al (2007) [^7^](#_ENREF_7) | | | | | | | |
| Population based on US census data (2000). Number and duration of contacts vary with age and location. Household contacts last longer than community contacts and are fairly uniform with age whilst community contacts are fairly assortative. Transmission rates vary by age (0-4, 5-18, 19-64 and ≥65 years) according to an asymmetrical WAIFW matrix, but not by location (household, day-care centre, school, workplace, community, long term care facility). At weekends, duration of household and community contacts are doubled and no contacts occur elsewhere. | R_0_ = 2.7  Latent period = 2 days  Baseline clinical AR 32% overall (calibrated to age-specific ARs from 1957pandemic: 36% (0-4 year olds) 62% (5-18), 25% (19-64), 21% (≥65), 33% (overall)) | Prevalence of infection of 10%, 15% or 20% in children at an individual school; schools remained closed for 7, 14 or 21 days. | Children from affected schools mix according to weekend contact patterns (i.e. contacts in schools eliminated; contacts in households and community doubled). | Decreased by ~30% if schools are closed for 14 days when prevalence reaches 10%. | Decreased by ~1-18%, depending on threshold and duration of closure: greater effect at lower thresholds; effect of duration of closure less clear.  Less effective for lower values of R_0_. Slightly greater effect if baseline contact intensity in schools increases. | Peak occurs 1 week earlier if schools are closed for 14 days when prevalence reaches 10%, compared to the no intervention scenario; no results presented for longer durations of closure. | Slight increase (~1 week) if schools are closed for 14 days when prevalence reaches 10%. |
|  | | | | | | | |
|  | | | | | | | |
|  | | | | | | | |
| Cauchemez et al (2008) [^8^](#_ENREF_8) | | | | | | | |
| Based on French census data (1999); stratified by age into adults (≥18 years) and children (<18 years) with contact occurring in households, schools and community | R_0_ = 1.8 during term time and 1.4 during holidays (estimated from data)  Serial interval = 2.4 days  Baseline clinical AR 31% (37-38% in children) | Daily incidence (all ages) ~20/100 000 or up to 1500/100000; schools closed permanently. | 24 % reduction of child-to-child transmission, no effect on adults’ contacts (based on analysis of French sentinel surveillance data covering term time and school holidays) | Decreased by 39-45% (47-52% in children). Reductions were smaller than this if schools closed at a higher threshold, e.g. 21% if threshold was 100 / 100,000 / day | Decreased by 13-17% (18-23% in children); greater reduction if schools closed at lower threshold. Reductions were smaller than this if schools closed at a higher threshold, e.g. 10% if threshold was 100 / 100,000 / day | NA | NA |
| Ciofi degli Atti et al (2008) [^9^](#_ENREF_9) | | | | | | | |
| Based on Italian census data (2001); structured into households, workplaces, day-care centres, schools, university and community. IBM coupled to SEIR model of global transmission. | R_0_ = 1.4, 1.7 or 2.0  Baseline cumulative clinical AR 21.2%, 30.8% or 38.7%, depending on R_0_.  Latent period = 1.5 days  Infectious period = 1.5 days (in SEIR model)  Mean serial interval = 2.6 days (in individual-based model) | Four weeks after first 20 symptomatic cases in the individual-based model; schools remain closed for 4 weeks. All schools and some non-essential public offices closed. | Not stated. | No appreciable effect | No appreciable effect | Increased by 5-8 days (2.5-8.8%) depending on transmissibility (greater delay for higher R_0_) | NA |
|  | | | | | | | |
| Yasuda et al (2008) [^10^](#_ENREF_10) | | | | | | | |
| Based on Japanese census data (date not stated), related statistical data for Tokyo and its suburbs, and time use data.  Probability of infection greatest in trains, then homes, then schools, then companies / stores. | Latent period = 2 days  “Period of infection” = 5 days  Baseline infection AR = 33% | 1-4 weeks after start of epidemic; schools remained closed for 2 weeks. | Not stated. | Decreased by ~23% if schools closed after 1-3 weeks, or by ~38% if schools closed after 4 weeks. | Changed by <10% for all closure thresholds. | If schools were closed 1-2 weeks after the start of the epidemic, peak delayed by 2-3 weeks; otherwise the epidemic curve became bimodal, with the larger peak occurring 3 weeks after (if schools closed after 3 weeks) or 1 week before (if closed after 4 weeks) the peak for the unmitigated epidemic. | Increased by ~4 weeks for all closure thresholds. |
|  | | | | | | | |
| Mniszewski et al (2008)[^11^](#_ENREF_11) | | | | | | | |
| Population based on census data for southern California (2000).  School closure modelled in combination with antiviral treatment / household prophylaxis from start of epidemic, and vaccination from 5 months (overlapping with the period of school closure by ~2 months) | Mean incubation period 1.9 days, “slightly longer than the latent period.”  Mean infectious period 4.1 days  Infection rate in children double that in adults  Baseline clinical AR 30.6%  R_0_ = 1.8 | Prevalence of symptomatic infection 0.1%; schools remain closed for six months | Children spend the time they would have spent at school at home instead; their other activities are not affected. | First wave peak AR decreased by ~98%; second wave peak AR 50-100% smaller than the unmitigated single peak, depending on vaccine properties. | Total AR (first and second waves) reduced by 28-96%, depending on vaccine properties | Reduced by ~1 week (for peak of first wave). | First wave duration increased by ~40 days; second wave may begin ~6 months after the end of the first and last for ~90 days |
|  | | | | | | | |
| Milne et al (2008) [^12^](#_ENREF_12) | | | | | | | |
| Population based on Australian census data (2001).  43% of infections occur in households, 29% in schools / workplaces, 26% in community.  Model incorporates simultaneous closure of schools, childcare facilities and adult education institutions | R_0_ = 1.5, 2.0 or 2.5  Latent period = 1 day  Infectious period = 5 days (including 1 day asymptomatic), or 3 or 8 days in sensitivity analyses  Baseline clinical attack rates 33%, 55% or 65%. Age-specific ARs calibrated against seasonal influenza data from Tecumseh, or 1968 pandemic. | Before the appearance of the first case; continued indefinitely. | School contacts eliminated; students and teachers spend the day at home so household contacts increase if others are present in the household. No effect on community contacts. If a child would otherwise be at home alone, an adult from the household stays at home and their workplace contacts are eliminated. | Reduced by 32-78%, depending on R_0_ (greater reduction for lower R_0_) | Decreased by 8-61%, depending on R_0_ (greater reduction for lower R_0_). Reduction of 59% if attack rates vary little with age, R_0_ = 1.5 and 38%, 29% and 32% of transmissions occur in households, schools / workplaces and community, respectively. | NA | NA |
|  | | | | | | | |
| Kelso et al (2009) [^13^](#_ENREF_13) | | | | | | | |
| As [^12^](#_ENREF_12), with emphasis on the timing of closing schools. | R_0_ = 1.5, 2.5 or 3.5  Latent period = 1 day  Infectious period = 5 days (including 1 day asymptomatic), or 3 or 8 days in sensitivity analyses  Baseline clinical attack rates 33%, 65% or 73%. For R_0_ = 1.5, baseline clinical ARs ~ 58% (0-5, 6-12 and 13-17 years), 44% (18-24 years), 40% (25-44 years) 25% (45-64years, ≥65 years), 33% (overall). | 0-8 weeks after appearance of first infectious case; continued indefinitely. | As [^12^](#_ENREF_12) | If R_0_=1.5, decreased by ~80% if delay is up to 4 weeks; benefit decreases for longer delays, with essentially no reduction for a delay of 8 weeks. If R_0_=2.5, decreased by ~33% for delays of 3 weeks or less, little effect if delay is 4 weeks or more. | If R_0_=1.5, reduced by ~60% if delay is up to 3 weeks; benefit decreases for longer delays, with a reduction of ~22% for a delay of 8 weeks. For R_0_ = 1.5 and pre-emptive closure, reductions in cumulative AR were ~57% (0-5 years), 64% (6-12 years) 66% (13-17 years), 57% (18-24 years), 58% (25-44 years), 56% (45-64 years), 52% (≥65 years).  If R_0_=2.5, reduction is <10% even if closures are implemented without delay. | If R_0_=1.5, delayed by ~17 days for delays up to 4 weeks; longer delays bring the peak forward by up to ~16 days. If R_0_=2.5, peak is delayed 5-12 days if closure is pre-emptive or within 2 weeks, otherwise little effect. | If R_0_=1.5, increased by up to ~30 days; if R_0_=2.5, increased by up to ~10 days. |
|  | | | | | | | |
|  | | | | | | | |
|  | | | | | | | |
| Sander et al (2009) [^14^](#_ENREF_14) | | | | | | | |
| Population based on US census data (2000). Structured into  households, neighbourhood clusters, neighbourhoods, playgroups, day-care centres, elementary, middle and high schools, workplaces, and the community. | Baseline clinical AR = 50% | Implied that schools are closed immediately at the start of the pandemic; remain closed for 26 weeks (the duration of the pandemic) | Not clear | NA | Decreased by 22% (from 50% to 39%). | NA | NA |
| Timpka et al (2009) [^15^](#_ENREF_15) | | | | | | | |
| Based on Swedish population (year not stated). Transmission probabilities highest in households, then schools, then community. | R_0_ = 2.23  Incubation period 1.9 days, infectious period 4.1 days.  Baseline AR not stated. | Not stated. | Contact at school eliminated, apparently no effects on other contact although this is not explicit.  No empirical basis stated for these assumptions. | NA | NA | NA | NA |
|  | | | | | | | |
| Sypsa & Hatzakis (2009) [^16^](#_ENREF_16) | | | | | | | |
| Based on Greek population (2001).  Transmission probabilities greatest in households, then schools, then neighbourhoods, then community. | R_0_ = 1.51 (estimated from model)  Latent period = 1 day  Infectious period = 4 days  Transmission probabilities based on previous model[^17^](#_ENREF_17) and modified to reflect data from 2009 H1N1 outbreak in La Gloria, Mexico.  Baseline clinical AR 34.5% overall (59.7% in 0-18 year olds, 32.1% in 19-65, 23.8% in ≥65) | Cumulative clinical attack rate of 1%. | Unclear; presumably school contacts are eliminated (100% of schools shut). 60% of children comply and stay at home, it is not explicit how this affects their contact patterns compared to being at school or to not complying. | NA | Reduced by 89% | NA | Shortened by 11 days |
|  | | | | | | | |
| Yasuda & Suzuki (2009) [^18^](#_ENREF_18) | | | | | | | |
| As [^10^](#_ENREF_10); probability of infection in school altered to be consistent with values estimated for H1N1v outbreaks amongst children in non-school settings. School closures modelled in combination with self-isolation of all student cases and 1/3 of adult cases. | Latent period = 2 days; “Period of infection” = 5 days.  Baseline infection AR = 36% | One or two weeks after start of outbreak, lasting for 4-7 days. | Not clear | Effects ranged from a decrease of 26% to an increase of 3%, depending on timing and duration of closure (compared to scenario with self-isolation alone): the greatest reduction was associated with the earlier closure (1 week after the start of the outbreak) but duration of closure had little effect if it was 5 days or greater. | Ranged from an increase of 0.7% to a decrease of 17%, depending on timing and duration of closure (compared to scenario with self-isolation alone): the greatest reductions were associated with the earlier closure (1 week after the start of the outbreak) and longest duration of closure. | Delayed by 1-2 weeks, depending on timing and duration of closure (compared to scenario with self-isolation alone). | Little or no apparent effect. |
|  | | | | | | | |
| Lee et al (2009) [^19^](#_ENREF_19) | | | | | | | |
| Based on census data for Allegheny County, Pennsylvania, USA (2000). Transmission parameters based on previous models [^5^](#_ENREF_5)^,^ [^20^](#_ENREF_20)^,^ [^21^](#_ENREF_21) and greatest in households, then workplaces, then schools, then community. | R_0_ = 1.4, 1.7, 1.9 or 2.4  Baseline infection AR ranged from 35.1% (if R_0_ = 1.4) to 53% (if R_0_ = 2.4).  Latent and infectious periods not stated. | Threshold prevalence of symptomatic cases in population of 0.1%, 0.5%, 1.0% or 1.5% if whole school systems are closed; or one day after the occurrence of 1, 5 or 10 symptomatic cases in the school if individual schools are closed.  Duration of closure varied from 1 to 16 weeks. | Contacts at school eliminated, no effect on community contacts. | Ranged from a reduction of 63.2% (if R_0_ was 1.4 and individual schools were closed for 16 weeks at a threshold of 1 case per school) to an increase of 9.2% (if R_0_ was 2.4 and individual schools closed for 1 week at a threshold of 1 case per school). No consistent differences between reductions predicted with closure of individual schools compared to the whole school system. | Ranged from a reduction of 44.7% (if R_0_ was 1.4 and individual schools were closed for 16 weeks at a threshold of 1 case per school) to an increase of 1.7% (if R_0_ was 1.7 and the whole school system was closed for 1 week at a threshold prevalence of 1% of the population). No consistent differences between reductions predicted with closure of individual schools compared to the whole school system. | Could be delayed by up to 28 days if R_0_ = 1.4 and whole school system is closed for 8 weeks at a threshold prevalence of 1% or less; other scenarios suggested shorter or no delays to the peak. | Difficult to assess precisely from graphs presented, but suggests an increase is likely (~10-20 days). |
|  | | | | | | | |
| Chao et al (2010) [^22^](#_ENREF_22) | | | | | | | |
| Population based on metropolitan Seattle. | R_0_ = 2.0  Baseline clinical AR 33%  Infectious period = 6 days, beginning one day after becoming infected.  Incubation period 1-3 days. | Threshold not stated; schools closed either for 60 days or permanently. | Contacts at school eliminated, household contacts increased by an unspecified amount, community contacts doubled. | Peak prevalence reduced by ~67% if schools closed permanently; if schools reopened after 60 days, epidemic was bimodal, with the first and second peaks in prevalence ~33% and 50% the size of the peak in the unmitigated case, respectively. | NA | Peak prevalence delayed by ~24 days; the second peak occurs ~10 days later (when schools are closed for 60 days). | Increased by ~90 days. |
| Chao et al (2011) [^23^](#_ENREF_23) | | | | | | | |
| Model as in Chao et al (2010) [^22^](#_ENREF_22), adapted for H1N1 pandemic in LA County (primary studies vaccination). | R_0_ = 1.3  Baseline infection AR 23%  Generation interval 3.4 days | For local, individual school closures, each school closed for 7 days following one case in the school.  For county-wide school closure, all schools in the county close for 7 days at an unspecified threshold | Not stated. | Peak prevalence reduced by ~5% by county-wide closures or ~26% by local closures. | Both strategies “did not elicit any substantive decrease” (this is not quantified further). | County-wide closures delayed the peak by ~1 week; local closures by ~4-5 weeks. | County-wide closures had little effect on duration; local closures increased the duration of the epidemic but it is not clear by how much. |
|  | | | | | | | |
| Halder et al (2010) [^24^](#_ENREF_24) | | | | | | | |
| As [^12^](#_ENREF_12) with emphasis on 2009 pandemic | R_0_ = 1.5  Baseline clinical AR 32.5%.  Mean infectious period = 5.5 days (including 1 day asymptomatic),  Mean generation time ~2.5 days | 1 case in a class triggers isolation of that case and their class (“school case isolation” strategy);  1 case in a primary school triggers closure of that school; 1 case in a secondary school triggers isolation of that class while 2 cases trigger full closure (“individual school closure” strategy);  30 symptomatic cases in the community (0.1% of the population) trigger closure of all schools (“all school closures” strategy).  Closure lasted 1-4 weeks in all cases. | School contacts eliminated; students and teachers spend the day at home so household contacts increase if others are present in the household. No effect on community contacts. If a child aged 5-12 would otherwise be at home alone, an adult from the household stays at home. | Reduced by ~13% (school case isolation), ~23% (individual school closure) or ~7% (all school closure) if closed for 1 week; individual school closure resulted in greater reductions with longer periods of closure (e.g. ~63% with 4 week closure) | Reduced by ~8% (school case isolation or individual school closure) or ~2% (all school closure) if closed for 1 week; individual school closure resulted in greater reductions with longer periods of closure (e.g. ~23% with 4 week closure) | No apparent effect of school case isolation; individual or all school closure delayed peak by ~10 days | Possible slight increase of ~10 days for all strategies. |
|  | | | | | | | |
| Kelso et al (2010) [^25^](#_ENREF_25) | | | | | | | |
| As [^12^](#_ENREF_12) with emphasis on antiviral treatment strategies and delays. Compares various antiviral treatment / prophylaxis strategies with and without school closures. | R_0_ = 1.5 (or 1.2, 2.0 or 2.5 in sensitivity analyses)  Baseline clinical AR 24.5%  Serial interval 2.32 days | Two diagnosed cases in a school triggers closure of that school for two weeks. | School contacts eliminated; students and teachers spend the day at home so household contacts increase if others are present in the household. No effect on community contacts. If a child aged 5-12 would otherwise be at home alone, an adult from the household stays at home. | For each antiviral strategy, adding school closure reduced the peak incidence by up to 50% compared to using antivirals alone (assuming no delay in diagnosis; effects decreased as delay increased). | For each antiviral strategy, adding school closure reduced the cumulative AR by ~20-30% compared to using antivirals alone (assuming no delay in diagnosis; effects decreased as delay increased). | Delayed by ~40 days for each antiviral strategy. | Increased by up to 40 days, depending on antiviral strategy. |
|  | | | | | | | |
| Halder et al (2010) [^26^](#_ENREF_26) | | | | | | | |
| As [^12^](#_ENREF_12) with emphasis on timing and duration of closure. | R_0_ = 1.5, 2.0 or 2.5.  Baseline clinical attack rates 33%, 50% and 59% for the respective values of R_0_.  Serial interval 2.49, 2.36 or 2.21 days. | Individual school closure strategy: 1 case in a primary school triggers closure of that school; 1-2 cases in a high school leads to isolation of students in the affected class; >2 cases in a high school triggers closure of the whole school. Closures occurred only when daily incidence in the community exceeded threshold levels between 0.003% and 0.333%.  Simultaneous school closure strategy: all schools close when incidence in the community exceeds the thresholds above. | As [^12^](#_ENREF_12). | Maximum reduction of 73% (R_0_ = 1.5) or 38% (R_0_ = 2.5), depending on timing and duration of closure.  Optimal threshold depended non-linearly on duration of closure. | Maximum reduction of 42% (R_0_ = 1.5), 18% (R_0_ = 2.0), 8% (R_0_ = 2.5) depending on timing and duration of closure.  Optimal threshold depended non-linearly on duration of closure. | Maximum delay ~45 days (if R_0_ = 1.5, schools closed for 8 weeks and closure was optimally timed). Smaller delays were possible with higher values of R_0_. | Markedly increased, particularly for low values of R_0_. |
| Barrett et al (2011) [^27^](#_ENREF_27) | | | | | | | |
| Population of 150,000 based on US census data; activities based on time use data (year of collection not stated). | Baseline attack rate (including individual preventive measures) 26.3%. | School close when “1% of the total population is infected”; closure lasts for two weeks. School closure is modelled in conjunction with personal preventive behaviours (e.g. buying over the counter antiviral medication and avoiding unnecessary trips), which vary with age and socioeconomic group. | Contacts at school appear to be eliminated although other contacts between children are not: 75% of children stay at home throughout closure whilst 25% continue their usual after-school activities. If a child aged ≤13 years would otherwise be alone at home, an adult stays at home to care for them. | Peak prevalence in children reduced by ~78% compared to the scenario with preventive behaviours only. No clear effect for adults or elderly. | Reduced by 40% compared to the scenario with preventive behaviours only. | Epidemic becomes bimodal. For children, peaks with school closure occur ~14 days before and ~3 days after the peak in the scenario with preventive behaviours only. No clear effect in adults; peak brought forward by ~3 days in elderly. | Shortened by ~20 days in children, adults and elderly. |
| Andradóttir et al (2011) [^28^](#_ENREF_28) | | | | | | | |
| Population based on Hamilton, Ontario, using Canadian census data from 2001 and 2006. | R_0_ = 1.4  Mean latent period 1.9 days; mean infectious period 4.1 days.  Baseline clinical attack rate 34.1%. | Each school closes for 5 days if ≥5 cases are identified in that school. | Not stated.  School closure modelled in conjunction with a reduction of 20% in contacts in workplaces and the general community. | NA | Reduced by 30% overall. Effect largest in adults (40% reduction) and smallest in schoolchildren (22% reduction). | NA | NA |
|  | | | | | | | |
| Yang et al (2011) [^29^](#_ENREF_29) | | | | | | | |
| Population based on Eemnes, The Netherlands (population 8382). Behaviour based on time use data, data on household size and land use data (further details not specified). | R_0_ = 1.79  Latent period 1-3 days  Infectious period 3-6 days  Baseline attack rate 68% | Schools closed the day after prevalence reaches 20 infections in the population. | Contacts at school eliminated; effects on other contacts unclear. | Reduced by 28.9% | Reduced by 4.2% | Delayed by 8 days | Increased by ~2 weeks |
| Zhang et al (2011) [^30^](#_ENREF_30) | | | | | | | |
| Based on population of Singapore, including location-specific numbers of contacts collected in a survey in 2008 (details of the collection of these data and the definition of contact are not provided) | R_0_ = 1.9 (or 1.5 or 2.3 in sensitivity analyses)  Mean generation time 2.5 days  Baseline clinical AR 44% | Schools closed when prevalence reached 0.02, 0.25, 1.5 or 5%, for a duration of 2, 4, 6, 8 or 10 weeks. Thresholds of 10 and 15% also investigated for closure lasting 2 weeks. | Contacts at school eliminated, no change in contacts occurring elsewhere. | Reduced by ~0-27% depending on threshold and duration of closure. Increasing duration of closure has little effect if it is 4 weeks or longer. Increasing threshold has little effect if it is ≤1.5% and duration is ≥4 weeks. | Reduced by <10% for all combinations of closure threshold and duration. Larger reductions as duration of closure increased up to 6 weeks, little additional benefit (and slight increase in cumulative AR if closure occurred late) to increasing duration beyond this unless the threshold was 0.02%. | Delayed by up to 5 days | NA |
| Mao (2011) [^31^](#_ENREF_31) | | | | | | | |
| Population based on US census data (2000) for Buffalo, New York. School closure modelled in combination with closure of 10% or 33% of workplaces. | R_0_ = 1.3 – 1.4  Average latent period 2 days  Infectious period 4-7 days  Baseline clinical attack rate 18.6% | Schools closed when cumulative number of symptomatic infections exceeds 1000 (~1% of population). | Contacts at school presumably eliminated, effects on other contacts not stated. | Reduced by ~63% if 10% of workplaces close or ~85% if 33% of workplaces close. | Reduced by 36% if 10% of workplaces close, or 74% if 33% of workplaces close. | Delayed by 3 days if 10% of workplaces close; brought forward by 8 days if 33% of workplaces close. | Increased by ~30 days |
| Morimoto & Ishikawa (2010) [^32^](#_ENREF_32) | | | | | | | |
| Population based on Sapporo city, Hokkaido, Japan, using census and other data from 2000, 2005 and 2007. | R_0_ = 2  Baseline infection attack rate 58% | All schools in a ward closed the day following diagnosis of an individual in that ward. | Not stated. | Reduced by 48% | Reduced by 14% | Delayed by 45 days. | Increased by ~70 days |
| Halder et al (2011) [^33^](#_ENREF_33) | | | | | | | |
| As [^12^](#_ENREF_12), with an emphasis on cost-effectiveness of interventions during a pandemic with characteristics similar to that of 2009. | R_n_ = 1.2, 1.5 or 1.8  Baseline clinical attack rate 13%, ~25% or ~33% for the respective values of R_n_. | As [^24^](#_ENREF_24); schools are closed for 2, 4 or 8 weeks or permanently. | As [^24^](#_ENREF_24) | NA | Reduced by 35-75% if R_n_ = 1.2, ~28-64% if R_n_ = 1.5, or ~18-42% if R_n_ = 1.8. Larger reductions with longer duration of closure. | NA | NA |
|  | | | | | | | |
| Zhang et al (2012) [^34^](#_ENREF_34) | | | | | | | |
| Population structure based on Singapore population data. School closure modelled in isolation or (primarily) in combination with partial closure of workplaces. | R_0_ = 1.9 (1.5 or 2.3 in sensitivity analyses)  Mean generation time 2.5 days  Baseline clinical attack rate 44% | Schools close at a threshold incidence of either 0.02%, 0.25%, 1.5% or 5%, in the whole population.  Schools remain closed for 2, 4, 6, 8 or 10 weeks. | Contacts at school eliminated; no effect on contacts elsewhere. Baseline contact patterns were based on a contact survey, but details are not given and the empirical data do not appear to include the effects of school closures. | Decreased by up to 28% by school closure alone. | Decreased by up to 9% by school closure alone. | Peak delayed by 5 days by school closure alone. | NA |
| **Network models** | | | | | | | |
| Carrat et al (2006) [^35^](#_ENREF_35) | | | | | | | |
| Based on French census data (year not stated). Networks within households, schools, workplaces, nursing homes and districts. | Average baseline clinical AR 33% overall (54% in 0-18 year olds, 28% in 19-65 year olds, 25% in >65s).  R_0_ estimated as 2.07 and serial interval as 2.44 days.  Latent period 0.5 days  Infectious period up to 10 days, peak infectiousness 2-3 days post-infection. | 5 infections / 1000 over an unspecified time period.  Schools reopened 10 days after the last observed infection. | Not explicit | Decreased by ~90% if only schools closed, or by ~97% if schools and workplaces closed. | Decreased by 79% if only schools closed (87% in children, 75% in adults, 76% in elderly), or by 98% if schools and workplaces closed (97% in children, 98% in adults, 97% in elderly). | No appreciable effect if only schools closed; peak is ~25 days earlier if schools and workplaces are closed. | Increased by ~30% if only schools are closed, or reduced by ~60% if schools and workplaces are closed. |
|  | | | | | | | |
| Glass et al (2006) [^36^](#_ENREF_36) | | | | | | | |
| Based on US census data (2000).  Household networks with mean link contact frequency (MLCF) 6/day; extended family / neighbourhood network with mean of 12.5 members and MLCF 1/day; school class network(s) of 20-35 with MLCF 6/day (children) or 1/day (teenagers); workplace network of 10-50 with MLCF 1/day; networks for gatherings of 5-20 older adults with MLCF 1/day; random links (3 in same age class, MLCF 1/day, across all ages MLCF 0.04/day. | R_0_=1.6 (estimated from model)  Mean latent period = 1.25 days  Infectious period = 2 days (including 0.5 days pre-symptomatic)  Baseline clinical AR 25% overall (39% in children, 36% in teenagers, 22% in adults, 12% in older adults). | 90% or 50% of schools closed the day following the 10^th^ symptomatic case. | Elimination of contacts at school, doubling of household contacts with or without doubling of other non-school contacts. Also assessed keeping children and teenagers at home for the duration of the pandemic.  No empirical basis stated for these assumptions. | Ranged from a reduction (from baseline of 7%) of 94% if children and teenagers were kept at home and compliance was 90%, to an increase of 27% if non-school contacts were doubled and compliance was 90% | Ranged from a reduction of 93% if children and teenagers were kept at home and compliance was 90%, to an increase of 18% if non-school contacts were doubled and compliance was 90% | Ranged from a reduction of 19 days if children and teenagers were kept at home and compliance was 90% to an increase of 15 days if children only were kept at home and compliance was 90% | Ranged from a reduction of 20 days  if children and teenagers were kept at home and compliance was 90% to an increase of 59 days if children and teenagers were kept at home and compliance was 50% |
|  | | | | | | | |
|  | | | | | | | |
|  | | | | | | | |
|  | | | | | | | |
|  | | | | | | | |
|  | | | | | | | |
| Davey & Glass (2008) [^37^](#_ENREF_37) | | | | | | | |
| As [^36^](#_ENREF_36), with emphasis on rescinding control measures | R_0_ = 1.6 or 2.0.  Latent period = 1.5-2 days  Infectious period = 1.5-2 days  Baseline clinical AR 25% or 36%, depending on R_0_ | 10 diagnosed cases within the community. Schools re-opened when 0, 1, 2 or 3 cases occur in 7 days and may be re-closed if the threshold is subsequently breached again; alternatively, schools remain closed for the duration of the epidemic. | Contact rates outside households reduced by specified compliance level (50-90%) for children, teenagers and one adult per household with children (children are “sequestered” in the home). Household contact frequencies doubled for these individuals.  No empirical basis stated for these assumptions. | Reduced by 53-95% depending on compliance and R_0_: for a given R_0_, the reduction increases with the compliance level; for a given compliance level, the reduction increases as R_0_ decreases.  Only fairly weak dependence on rescinding threshold. | Reduced by 21-96% depending on compliance, rescinding threshold and R_0_: reduction increases with increasing compliance, decreasing R_0_ and decreasing rescinding threshold. | NA | NA |
| Davey et al (2008) [^38^](#_ENREF_38) | | | | | | | |
| As [^36^](#_ENREF_36), exploring a wider range of scenarios | ”Scaled disease infectivity” 0.75 – 3.0 (a value of 1.5 is equivalent to R_0_ ~ 2).  Latent period = 1.25 days  Infectious period = 2 days (including 0.5 days pre-symptomatic)  Baseline cumulative clinical AR 14-46%, depending on infectivity | Introduced at cumulative incidence of 10, 30 or 100 diagnosed, symptomatic cases with compliance of 60% or 90%; maintained until the number of new cases in 7 days reaches 3 or 0. Schools can re-close if the threshold is subsequently reached again. | All school contacts reduced by 90%. Household contacts doubled for affected children. One adult stays at home in each household with a child <11 years.  No empirical basis stated for these assumptions. | NA | Reduction ranged from 2% (for infectivity factor of 3.00) to 92% (for infectivity factor of 0.75). | NA | NA |
| Perlroth et al (2010) [^39^](#_ENREF_39) | | | | | | | |
| As [^36^](#_ENREF_36), with focus on economic aspects. | R_0_ = 2.1 or 1.6  Mean infectious period 1.5 days  Baseline clinical AR ~25% (if R_0_ = 1.6) or ~35% (if R_0_ = 2.1) | Introduced when 10 people (0.0001% of the population) have become symptomatic; schools reopen after 2 generation times have passed without new cases being diagnosed. | School contacts reduced by 90%, children’s household contacts doubled | NA | Reduced by 66% (if R_0_ = 1.6) or 12% (R_0_ = 2.1). | NA | NA |
| **Compartmental models** | | | | | | | |
| Roberts et al (2007) [^40^](#_ENREF_40) | | | | | | | |
| SEIR model structured by location (household, schools, workplaces and community) but not explicitly by age. 47% of infections occur in households, 24% in schools, 18% in workplaces and 11% in community. | R_0_ = 1.1, 2.0 or 3.0  Latent period = 1.2, 1.6 or 2.0 days  Infectious period = 4.1 days  Baseline clinical AR = 12%, 53% or 63%, for R_0_ = 1.1, 2.0 and 3.0, respectively. | Schools closed immediately at start of epidemic | Elimination of transmission within schools; no effect on transmission in other locations.  No empirical basis stated for these assumptions. | NA | If R_0_ = 1.1, cumulative AR is close to zero (and R<1) if transmission in schools is reduced by 37%. Cumulative AR reduced by 25% or 12% if R_0_ = 2.0 or 3.0 respectively. | NA | NA |
|  |  |  |  |  |  |  |  |
| Rizzo et al (2008) [^41^](#_ENREF_41) | | | | | | | |
| Age / location- and region-structured SEIR model including stochastic component; contact matrix defined by location (households, schools / workplaces, community). Age groups 0-2, 3-14, 19-39, 40-64 and ≥65 years. Population based on Italian census data (2001).  School closures (for 3 weeks) incorporated together with closure of public offices (for 4 weeks) and public meeting places (for 8 weeks). | R_0_ = 1.8  Incubation period = 1 day  Infectious period = 3.9 days  Baseline infected AR 35%. | 2,4 or 8 weeks after the start of the pandemic | 75% reduction in contacts among children and teenagers. Workplace closure reduces work-related contact by 16%, closure of public places reduces community contacts by 50%. | NA | Decreased by <1% if intervention implemented 2 or 4 weeks after start of pandemic, or by 2.6% if after 8 weeks. | NA | NA |
| Cruz-Pacheco et al (2009) [^42^](#_ENREF_42) | | | | | | | |
| SIR model assuming homogeneous mixing, used to estimate change in contact rate with introduction of control measures, based on fitting to data from the 2009 pandemic in Mexico City. | Infectious period = 3 days.  R_0_ estimated as 1.72 and 1.27 before and after introduction of control measures, respectively. | Controls (including school closures as well as other measures) introduced ~1 week after start of outbreak, with or without relaxation ~2 weeks later. | Reduced by 27% (based on reported values of R_0_ before and after introduction of control measures during the H1N1v outbreak in Mexico City) in a linear fashion over six days. | Peak prevalence reduced by 38% (from 10.5% to 6.5%) if control measures relaxed, reduced by 67% (to 3.5%) if not relaxed. | NA | Delayed by ~1 week. | Increased by 2-3 weeks if contact rate recovers instantaneously when controls are lifted. (Epidemic with no intervention lasts ~5 weeks) |
|  | | | | | | | |
| Vynnycky & Edmunds (2008) [^43^](#_ENREF_43) | | | | | | | |
| SEIR model with age-dependent contact rates based on several different WAIFW matrices and fitting to data from 1957 pandemic; stratified into <1, 1-4, 5-14, 25-44, 45-64, ≥65 year olds. | R_0_ = 1.5-3.5  Latent period = 1.5-2 days  Infectious period = 1.5-2 days | Schools and nurseries closed when overall disease incidence of 50, 100, 200 or 1000/100 000 per week; reopened when incidence declined below the threshold. | Decreased within-group contact rates for children aged 1-4 and 5-14 by 25-75%; no effect on other contact rates.  Based in part on previous estimates of weekly transmission parameters for measles[^44^](#_ENREF_44), which considered contacts between schoolchildren and compared school terms to school holidays. | Decreased by ~0-60%, depending on R_0_, baseline mixing patterns, reduction in contacts and closure threshold. Greatest reductions associated with greatest reductions in contact, least assortative mixing patterns, lowest threshold for closure, and low values of R_0_. | Decreased by <1% to ~24%, depending on R_0_, baseline mixing patterns, reduction in contacts and closure threshold.  Greatest reduction if assumed reduction in contact is large, mixing is least assortative, and R_0_ is low. | Delayed by 1-2 weeks if R_0_ = 1.8 or 2.5, contact reduction = 75%, and closure threshold = 1000/100,000 per week. Otherwise no effect. | Little or no effect for high R_0_ or if reduction in contact is ≤50%. If R_0_~1.8, increased by up to 70% and 40% if schools are closed early or late, respectively. |
|  | | | | | | | |
| House et al (2011) [^45^](#_ENREF_45) | | | | | | | |
| SEIR model stratified by age and risk group, based on population of England (data from 2008) and incorporating empirical contact data from Polymod.  Percentage reduction in peak demand for intensive care unit beds in each hospital assumed to be equal to the percentage of children in that hospitals’ catchment area who are affected by school closure. | R_0_ = 1.1, 1.4 or 2.0. | Schools close for 1-4 weeks within half a day of the optimal time point for minimising peak incidence. | Age-specific changes consistent with Polymod data. | Reduced by 30-70%; size of reduction increased with increasing duration of closure and increasing R_0_. | NA | NA | NA |
|  | | | | | | | |
| Araz et al (2012) [^1^](#_ENREF_1) | | | | | | | |
| Age-structured SEIR model based on Texas population data. | Average latent period 3 days  Average infectious period 6 days  R_0_ 1.1, 1.3 or 1.5 in low transmission scenario, 1.5, 1.8 or 2.1 in high transmission scenario (results are combined for different R_0_ values in each of these two scenarios) | Threshold prevalence of 0.5%, 0.8%, 1.1%, 1.4%, 1.7%, 2.0%, 3.0%, 4.0%, 5.0% or 6.0% in children aged 5-18 years. Closure lasted either for 1, 2, 3, 4, 8, 12 or 24 weeks, or until prevalence in 5-18 year olds had declined to 75%, 50% or 25% of the closure threshold. | Number of daily child-to-child contacts reduced by 80%. Contacts between adults and children either unaffected or reduced by 33%, depending on the direction of the contact. No effect on contact between adults. Assumptions based on Polymod contact data [^46^](#_ENREF_46). | Peak prevalence reduced by ~80% (low transmission scenario) or ~88% (high transmission scenario). | For low transmission scenario, reduction in cumulative AR was 5-94% in children aged 5-18 years and -37 to 78% in adults, depending on threshold and duration. Greatest effects with longest closures, but little additional benefit of closing for >12 weeks. If duration was over ~8 weeks, benefits were greatest with the lowest closure thresholds.  For high transmission scenario, reduction in cumulative AR was -3 to 86% for 5-18 year olds and -48 to 32% for adults. | Peak brought forward by ~60 days (low transmission scenario) or ~35 days (high transmission scenario). | Reduced by ≥75 days (low transmission scenario) or increased by ≥25 days (high transmission scenario). |
| Ghosh & Heffernan 2010 | | | | | | | |
| SEIR model which also includes the effects of antivirals and vaccination. Infectious individuals are subdivided into those who are asymptomatic, untreated symptomatic, early treated symptomatic and late treated symptomatic; the recovered compartment is subdivided in an analogous way. Two pandemic waves are modelled with and without assuming that the first wave coincides with school summer holidays. | R_0_ 1.6  Latent period 3 days  Infectious period 4.85 days | “School holidays are assumed to start 70 days after the first wave emerges and last approximately 60 days” (in the model without intervention, the peak occurs around day 60-70). | Transmission parameter reduced by 30%. | First wave: reduced by ~38%  Second wave: reduced by ~95% | First wave: reduced by ~45%  Second wave: reduced by ~77% | First wave: no effect  Second wave: delayed by ~50-60 days | First wave: no effect  Second wave: effect unclear. |
|  | | | | | | | |
| Earn et al (2012) [^47^](#_ENREF_47) | | | | | | | |
| SIR model with two age groups (5-18 year olds and others) fitted to pandemic H1N1 2009 data from Alberta, Calgary and Edmonton and used to predict the course of the epidemic if a planned school closure had not occurred. Transmission within each age group allowed to vary with temperature, absolute humidity and school closure. | R_0_ 1.72 – 1.78 (estimated value depending on dataset) | In the observed data, schools were closed for a planned holiday close to the beginning of the first wave; a model fitted to these data was used to predict how the epidemic might have developed had this school closure not occurred. | Transmission between children aged 5-18 years reduced by 63% (Calgary), 100% (Edmonton), 86% (Alberta), with no change in transmission amongst other age groups, based on fitting to the incidence data. | First wave, school aged children: reduced by ~70% in Alberta and Calgary, very little effect in Edmonton;  Other ages: reduced by ~79% in Alberta, ~71% in Calgary, very little effect in Edmonton. | Calgary: reduced by ~28%  Edmonton: reduced by ~35%  Alberta: reduced by ~52% | Delayed by ~1 month | Duration of first wave increased by up to ~1 month |
|  | | | | | | | |
| Bolton et al (2012) [^48^](#_ENREF_48) | | | | | | | |
| Stochastic patch model describing transmission between aimags in Mongolia (not age-structured). Movement between aimags (provinces) based on national travel statistics (year of collection not stated). | Mean latent period 1 day  Mean infectious period 1.5 days  R_0_ = 1.6 or 2.0.  Baseline clinical attack rate 9.7%. | Schools closed at times ranging from week 0 to week 14 of the pandemic, for 2-12 weeks. | School closure reduces the effective reproduction number by a factor equal to the ratio of the attack rate in adults to that in children; this ratio was allowed to vary between 0.3 and 1. | “Modest impact” (not quantified). | Maximum reduction of ~11% (if schools closed for 4 weeks starting from week 5 and attak rate in children was 3 times that in adults). Smaller reductions were predicted as attack rates in adults and children became more similar and as school closure was delayed beyond week 5. | Delayed by up to two weeks. | NA |
|  | | | | | | | |
| **Other models** | | | | | | | |
| Glass & Barnes (2007) [^49^](#_ENREF_49) | | | | | | | |
| Household model describing transmission within and between households, in the community and schools / workplaces.  Population based on Australian census data (2001). | R_0_ = 1.5 or 2.5  Serial interval = 3.5 days  Age-specific ARs calibrated to 1957 and 1968 pandemics. | Schools closed at start of outbreak, or at varying prevalence of infection in schoolchildren. | Elimination of transmission among children at school; increase in transmission between schoolchildren in households is increased in proportion to the extra time spent at home. Also allowed for one adult to stay home in every household with schoolchildren and no non-working adult. No effect on community mixing. | Decreased by ~10-70% depending on age-specific attack rates and R_0_: greater reduction for lower R_0_ and if attack rates are higher in children than in adults. Slightly greater reduction if parents stay home to look after children. | If schools are closed when prevalence in schoolchildren is 2%, decreased ~4-64% depending on age-specific attack rates and R_0_: greater reduction with lower R_0_ and if attack rates are higher in children than in adults.  Similar results if schools reopen as the epidemic declines, as long as closure occurs when prevalence in children is <1%. | Delayed by 1-15 weeks, depending on age-specific attack rates and R_0_: longer delay for lower R_0_ and if attack rates are higher in children than in adults. Delay increased if parents stay home to look after children. | Increased by 20-75% (1-3 weeks) depending on age-specific attack rates and R_0_: greater increase for lower R_0_ and if attack rates are higher in children than in adults. |

**References**

1. Araz, O.M., et al., *Simulating school closure policies for cost effective pandemic decision making.* BMC Public Health, 2012. **12**(1): p. 449.

2. Elveback, L.R., et al., *An influenza simulation model for immunization studies.* American Journal of Epidemiology, 1976. **103**(2): p. 152-65.

3. Ferguson, N.M., et al., *Strategies for containing an emerging influenza pandemic in Southeast Asia.* Nature, 2005. **437**(7056): p. 209-14.

4. Yasuda, H., N. Yoshizawa, and K. Suzuki, *Modeling on social spread from immunity.* Jpn J Infect Dis, 2005. **58**(6): p. S14-5.

5. Ferguson, N.M., et al., *Strategies for mitigating an influenza pandemic.* Nature, 2006. **442**(7101): p. 448-52.

6. Germann, T.C., et al., *Mitigation strategies for pandemic influenza in the United States.* Proceedings of the National Academy of Sciences of the United States of America, 2006. **103**(15): p. 5935-40.

7. Haber, M.J., et al., *Effectiveness of interventions to reduce contact rates during a simulated influenza pandemic.* Emerging Infectious Diseases, 2007. **13**(4): p. 581-9.

8. Cauchemez, S., et al., *Estimating the impact of school closure on influenza transmission from Sentinel data.* Nature, 2008. **452**(7188): p. 750-4.

9. Ciofi degli Atti, M.L., et al., *Mitigation measures for pandemic influenza in Italy: an individual based model considering different scenarios.* PLoS ONE [Electronic Resource], 2008. **3**(3): p. e1790.

10. Yasuda, H., et al., *Preparedness for the spread of influenza: prohibition of traffic, school closure, and vaccination of children in the commuter towns of Tokyo.* Journal of Urban Health, 2008. **85**(4): p. 619-35.

11. Mniszewski, S.M., et al., *Pandemic simulation of antivirals plus school closures: buying time until strain-specific vaccine is available.* Computational and Mathematical Organization Theory, 2008. **14**(3): p. 209-221.

12. Milne, G.J., et al., *A small community model for the transmission of infectious diseases: comparison of school closure as an intervention in individual-based models of an influenza pandemic.* PLoS ONE [Electronic Resource], 2008. **3**(12): p. e4005.

13. Kelso, J.K., G.J. Milne, and H. Kelly, *Simulation suggests that rapid activation of social distancing can arrest epidemic development due to a novel strain of influenza.* BMC Public Health, 2009. **9**: p. 117.

14. Sander, B., et al., *Economic Evaluation of Influenza Pandemic Mitigation Strategies in the United States Using a Stochastic Microsimulation Transmission Model.* Value Health, 2009. **12**(2): p. 226-233.

15. Timpka, T., et al., *Population-based simulations of influenza pandemics: validity and significance for public health policy.* Bulletin of the World Health Organization, 2009. **87**(4): p. 305-11.

16. Sypsa, V. and A. Hatzakis, *School closure is currently the main strategy to mitigate influenza A(H1N1)v: a modeling study.* Euro Surveillance: Bulletin Europeen sur les Maladies Transmissibles = European Communicable Disease Bulletin, 2009. **14**(24).

17. Longini, I.M., Jr., et al., *Containing pandemic influenza with antiviral agents.* American Journal of Epidemiology, 2004. **159**(7): p. 623-33.

18. Yasuda, H. and K. Suzuki, *Measures against transmission of pandemic H1N1 influenza in Japan in 2009: simulation model.* Euro Surveillance: Bulletin Europeen sur les Maladies Transmissibles = European Communicable Disease Bulletin, 2009. **14**(44).

19. Lee, B.Y., et al., *Simulating school closure strategies to mitigate an influenza epidemic.* Journal of Public Health Management & Practice, 2010. **16**(3): p. 252-61.

20. Halloran, M.E., et al., *Modeling targeted layered containment of an influenza pandemic in the United States.* Proceedings of the National Academy of Sciences of the United States of America, 2008. **105**(12): p. 4639-44.

21. Longini, I.M., Jr., et al., *Containing pandemic influenza at the source.* Science, 2005. **309**(5737): p. 1083-7.

22. Chao, D.L., et al., *FluTE, a publicly available stochastic influenza epidemic simulation model.* PLoS Computational Biology, 2010. **6**(1): p. e1000656.

23. Chao, D.L., et al., *Planning for the control of pandemic influenza A (H1N1) in Los Angeles County and the United States.* American Journal of Epidemiology, 2011. **173**(10): p. 1121-1130.

24. Halder, N., J.K. Kelso, and G.J. Milne, *Analysis of the effectiveness of interventions used during the 2009 A/H1N1 influenza pandemic.* BMC Public Health, 2010. **10**: p. 168.

25. Kelso, J.K., N. Halder, and G.J. Milne, *The impact of case diagnosis coverage and diagnosis delays on the effectiveness of antiviral strategies in mitigating pandemic influenza A/H1N1 2009.* PLoS ONE [Electronic Resource], 2010. **5**(11): p. e13797.

26. Halder, N., J.K. Kelso, and G.J. Milne, *Developing guidelines for school closure interventions to be used during a future influenza pandemic.* BMC Infectious Diseases, 2010. **10**: p. 221.

27. Barrett, C., et al., *Economic and social impact of influenza mitigation strategies by demographic class.* Epidemics, 2011. **3**(1): p. 19-31.

28. Andradottir, S., et al., *Reactive strategies for containing developing outbreaks of pandemic influenza.* BMC Public Health, 2011. **11 Suppl 1**: p. S1.

29. Yang, Y., P.M. Atkinson, and D. Ettema, *Analysis of CDC social control measures using an agent-based simulation of an influenza epidemic in a city.* BMC Infectious Diseases, 2011. **11**: p. 199.

30. Zhang, T., et al., *Temporal factors in school closure policy for mitigating the spread of influenza.* Journal of Public Health Policy, 2011. **32**(2): p. 180-97.

31. Mao, L., *Evaluating the combined effectiveness of influenza control strategies and human preventive behavior.* PLoS ONE [Electronic Resource], 2011. **6**(10): p. e24706.

32. Morimoto, T. and H. Ishikawa, *Assessment of intervention strategies against a novel influenza epidemic using an individual-based model.* Environmental Health and Preventive Medicine, 2010. **15**(3): p. 151-161.

33. Halder, N., J.K. Kelso, and G.J. Milne, *Cost-effective strategies for mitigating a future influenza pandemic with H1N1 2009 characteristics.* PLoS ONE [Electronic Resource], 2011. **6**(7): p. e22087.

34. Zhang, T., et al., *Evaluating temporal factors in combined interventions of workforce shift and school closure for mitigating the spread of influenza.* PLoS ONE [Electronic Resource], 2012. **7**(3): p. e32203.

35. Carrat, F., et al., *A 'small-world-like' model for comparing interventions aimed at preventing and controlling influenza pandemics.* BMC Medicine, 2006. **4**: p. 26.

36. Glass, R.J., et al., *Targeted social distancing design for pandemic influenza.* Emerging Infectious Diseases, 2006. **12**(11): p. 1671-81.

37. Davey, V.J. and R.J. Glass, *Rescinding community mitigation strategies in an influenza pandemic.* Emerging Infectious Diseases, 2008. **14**(3): p. 365-72.

38. Davey, V.J., et al., *Effective, robust design of community mitigation for pandemic influenza: a systematic examination of proposed US guidance.* PLoS ONE [Electronic Resource], 2008. **3**(7): p. e2606.

39. Perlroth, D.J., et al., *Health outcomes and costs of community mitigation strategies for an influenza pandemic in the United States.* Clinical Infectious Diseases, 2010. **50**(2): p. 165-74.

40. Roberts, M.G., et al., *A model for the spread and control of pandemic influenza in an isolated geographical region.* Journal of the Royal Society Interface, 2007. **4**(13): p. 325-30.

41. Rizzo, C., et al., *Scenarios of diffusion and control of an influenza pandemic in Italy.* Epidemiology & Infection, 2008. **136**(12): p. 1650-7.

42. Cruz-Pacheco, G., et al., *Modelling of the influenza A(H1N1)v outbreak in Mexico City, April-May 2009, with control sanitary measures.* Euro Surveillance: Bulletin Europeen sur les Maladies Transmissibles = European Communicable Disease Bulletin, 2009. **14**(26).

43. Vynnycky, E. and W.J. Edmunds, *Analyses of the 1957 (Asian) influenza pandemic in the United Kingdom and the impact of school closures.* Epidemiology & Infection, 2008. **136**(2): p. 166-79.

44. Fine, P.E. and J.A. Clarkson, *Measles in England and Wales--I: An analysis of factors underlying seasonal patterns.* International Journal of Epidemiology, 1982. **11**(1): p. 5-14.

45. House, T., et al., *Modelling the impact of local reactive school closures on critical care provision during an influenza pandemic.* Proceedings of the Royal Society of London - Series B: Biological Sciences, 2011. **278**(1719): p. 2753-60.

46. Mossong, J., et al., *Social contacts and mixing patterns relevant to the spread of infectious diseases.* PLoS Med, 2008. **5**(3): p. e74.

47. Earn, D.J., et al., *Effects of school closure on incidence of pandemic influenza in Alberta, Canada.[Summary for patients in Ann Intern Med. 2012 Feb 7;156(3):I28; PMID: 22312154].* Annals of Internal Medicine, 2012. **156**(3): p. 173-81.

48. Bolton, K.J., et al., *Likely effectiveness of pharmaceutical and non-pharmaceutical interventions for mitigating influenza virus transmission in Mongolia.* Bulletin of the World Health Organization, 2012. **90**(4): p. 264-71.

49. Glass, K. and B. Barnes, *How much would closing schools reduce transmission during an influenza pandemic?* Epidemiology, 2007. **18**(5): p. 623-8.
